# Free-floating thrombus in lower limb deep veins and May-Thurner syndrome: case report

**DOI:** 10.1590/1677-5449.200075

**Published:** 2020-11-16

**Authors:** Helio Bergantini, Selma Regina de Oliveira Raymundo, Daniel Gustavo Miquelin, Gleison Juliano da Silva Russeff, Desirée Francini del Frari Silva, Gabriela Leopoldino da Silva, Amabile Oficiati de Carnevale Galeti, Matheus Pietraroia dos Santos

**Affiliations:** 1 Faculdade de Medicina de São José do Rio Preto – FAMERP, São José do Rio Preto, SP, Brasil.; 2 Hospital Austa de São José do Rio Preto, São José do Rio Preto, SP, Brasil.

**Keywords:** venous thrombosis, anticoagulants, fibrinolysis, pulmonary embolism

## Abstract

Free-floating thrombus in the deep venous system has a high potential to cause pulmonary embolization. It can also be found in patients with superficial venous thrombosis (SVT) that extends to a deep vein. There are still no defined criteria for treatments described in the literature, which range from anticoagulation and fibrinolytic treatments with vena cava filter implants, through open or endovascular thrombectomies, to more invasive procedures such as surgical interruption with ligation of the venous system. We present the case of a patient with extensive deep venous thrombosis affecting the iliofemoral-popliteal territory with a floating thrombus extending from the left common iliac vein to the inferior vena cava. Treatment was performed with fibrinolytic therapy delivered with a multiperforated catheter, supplemented with anticoagulation with heparin and daily control angiography. At the end of the treatment, a significant stenosis was identified in the left common iliac vein, and angioplasty was performed with stenting.

## INTRODUCTION

More than three decades ago, venous thromboembolism (VTE) was estimated as the third most common acute cardiovascular event with an overall annual incidence of 1 to 2 per 1,000 people in the United States.^1^^-^3 Thrombi located in the deep vein system of the lower limbs constitute the most common source of embolization, primarily those in the iliac, femoral, and popliteal veins.^4^ May-Thurner Syndrome is another important element in genesis of diseases of the deep veins.

Free-floating venous thrombus (FFVT) is a specific subtype of deep venous thrombosis (DVT) with a high potential for pulmonary embolization.^5^^,^^6^ An FFVT exhibits continuous oscillatory movement of a thrombotic mass not adhered to the venous wall in regions of confluence of large veins: superficial-deep femoral, saphenous-femoral, internal-external iliac, and iliac-vena cava.^6^ An FFVT may also be present in patients with isolated superficial venous thromboses (SVT),^7^ which can possibly extend to a deep vein and, additionally, may be a complication of intravenous laser treatment of insufficient saphenous veins.^8^

Studies of FFVT are rare and divergent in terms of aspects such as mortality, prevalence, and predominant site of thrombi. Voet and Afschrift^9^ conducted a study of 44 cases of proximal DVT, reporting an 18% prevalence of FFVT with the following distribution: 38% at the saphenofemoral junction, 26% and the junction of the small saphenous vein, and 15% in the external iliac vein. These authors observed with duplex ultrasound (DUS) that 87% of the thrombi had disappeared after 3 months of anticoagulant treatment, irrespective of their site. In turn, Norris et al.^5^ studied 78 hospitalized patients with iliofemoral DVT diagnosed by venography, observing a notable difference in the risk of pulmonary embolization, confirmed by pulmonary ventilation/perfusion scintigraphy performed 10 days after venography, between occlusive thrombi (5.5%) and free-floating thrombi (60%). All patients were treated with heparin. In turn, Yamaki et al.^10^ documented a 1.7% rate of floating thrombi among 427 patients diagnosed with DVT, the majority (71.4%) located in the femoropopliteal segment. This elevated potential for complications is because FFVT typically form at the confluences of large vessels, compounded by their greater instability.^4^^-^6

With regard to treatment, a number of different approaches are described in the literature. Some studies suggest only anticoagulation, because of autolysis of floating thrombi, combined with rest.^9^^,^^10^ Others prescribe surgical treatment.^6^ We present a case of FFVT in the left common iliac vein (LCIV) treated with an endovascular procedure involving fibrinolysis, angioplasty, and stenting.

## CASE DESCRIPTION

**Figure 1:**
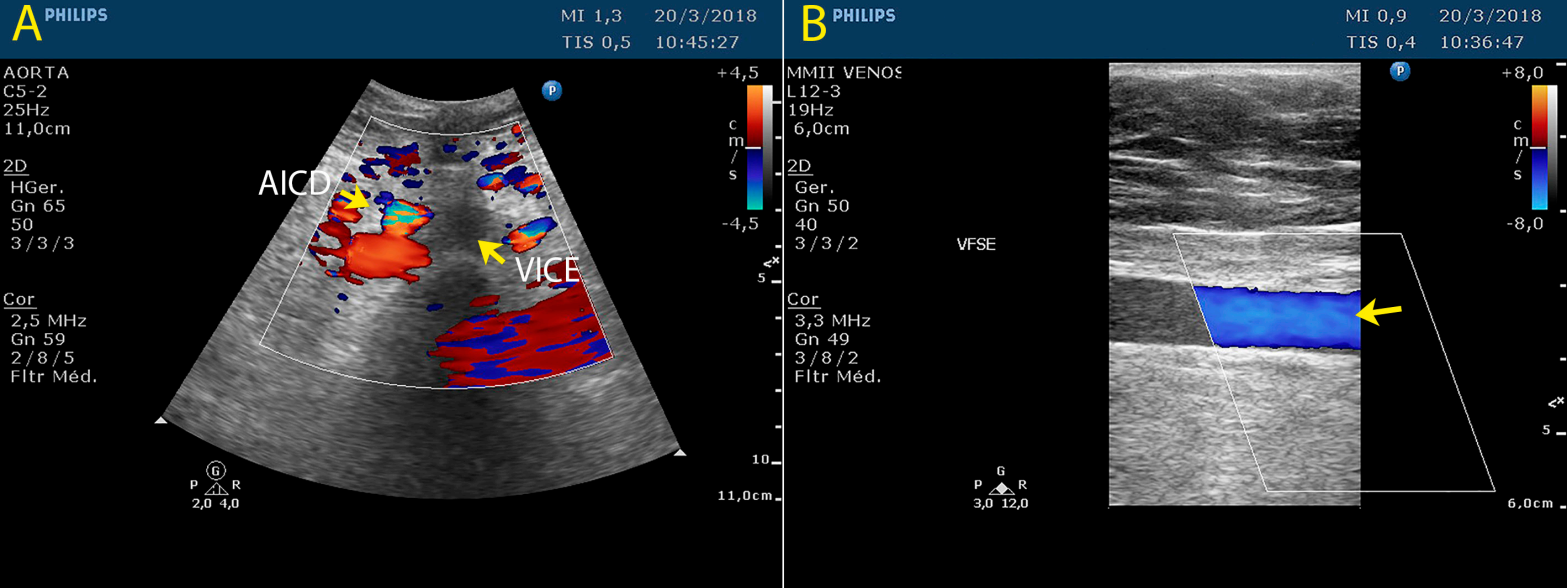
Duplex ultrasound (DUS) showing: (A) left common iliac vein (LCIV) with large caliber and no blood flow, with hypoechoic intraluminal material indicating acute deep venous thrombosis (DVT) and compression by the right common iliac artery (RCIA); and (B) left superficial femoral vein (LSFV) with normal caliber, flow present, and absence of thrombi.

The patient was a 47-year-old female who had been admitted with intense pain and edema in the left lower limb (LLL) the day before. Physical examination revealed edema of the limb starting at the thigh, clubbing, venous flushing, and the Homans and Bancroft signs. DUS showed enlarged calibers of the left common and external iliac veins, with no blood flow, and hypoechoic intraluminal material, indicating acute DVT. The femoral, popliteal, tibial, and fibular veins were compressible and had normal flow, with no evidence of thrombi (Figure 1).

Anticoagulation was initiated with full dosage low molecular weight heparin (LMWH). After 2 days of treatment, the patient’s edema worsened, and the decision was taken to perform thrombolysis.

**Figure 2:**
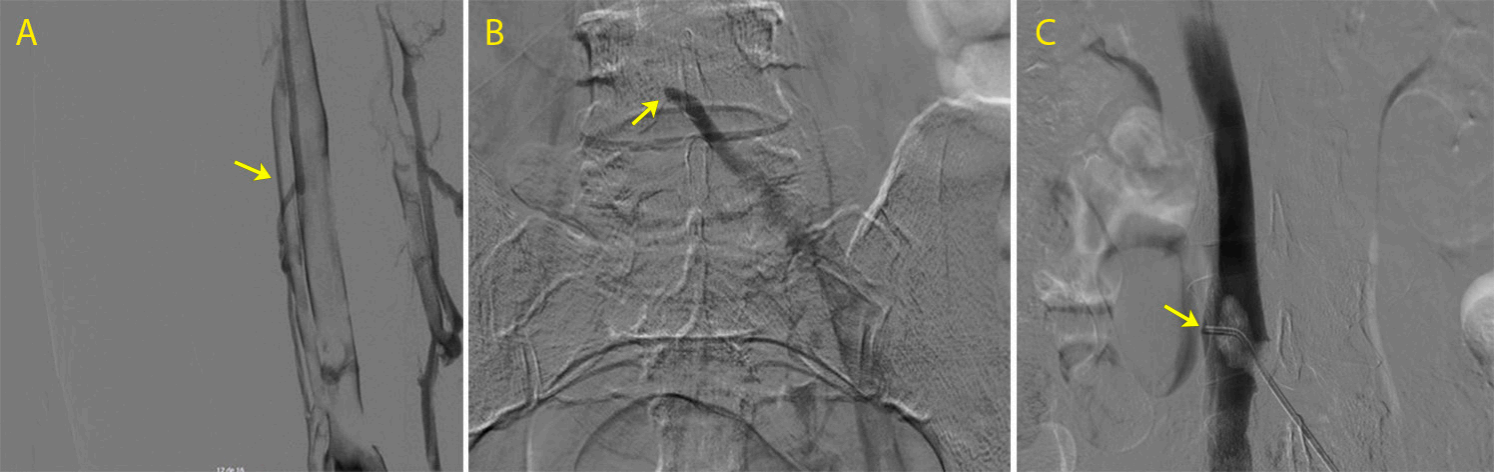
Phlebography showing: (A) progression of thrombosis to the femoral and popliteal veins; (B) extensive thrombosis of the left iliac veins and inferior vena cava (IVC); and (C) presence of free-floating thrombus in the IVC (arrow).

Phlebography was conducted via left popliteal vein DUS-guided puncture, using a 6Fr Bernstein catheter (AngioDynamics, Inc., New York, United States), showing progression of the thrombosis to the femoral and popliteal veins, which had not been present when the DUS examination had been conducted on the day of admission, as can be observed in Figure 1. Phlebography also showed a free-floating thrombus in the inferior vena cava (IVC) (Figure 2). For fibrinolysis, a Fountain infusion microcatheter (Merit Medical Systems, Inc., Utah, United States) was advanced up to the LCIV and 10 mg of Alteplase fibrinolytic was injected in bolus. The catheter was left in place for continuous infusion of the fibrinolytic at a dosage rate of 0.01 mg/kg/hour. Intravenous heparin was administered in continuous infusion simultaneously with the fibrinolytic.

**Figure 3:**
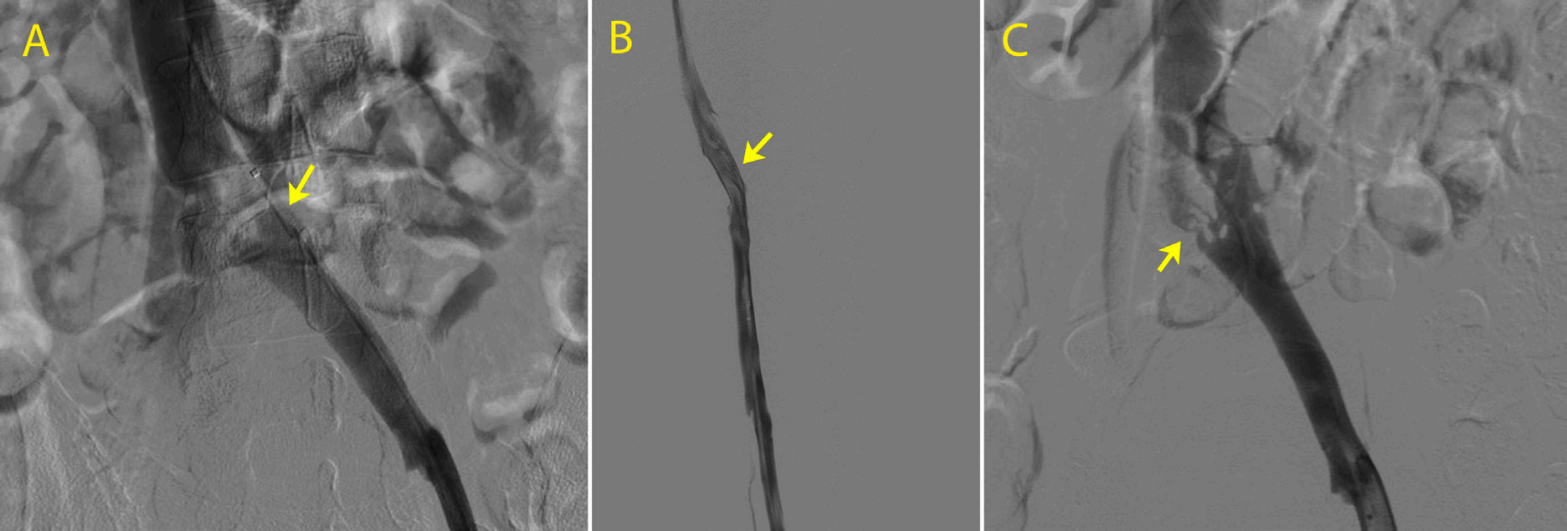
Control phlebography 72 hours after fibrinolysis showing: (A) recanalization of the inferior vena cava (IVC) and left common iliac vein (LCIV); (B) recanalization of the superficial femoral vein; and (C) extrinsic compression of the LCIV, characteristic of May-Thurner Syndrome (arrow).

**Figure 4:**
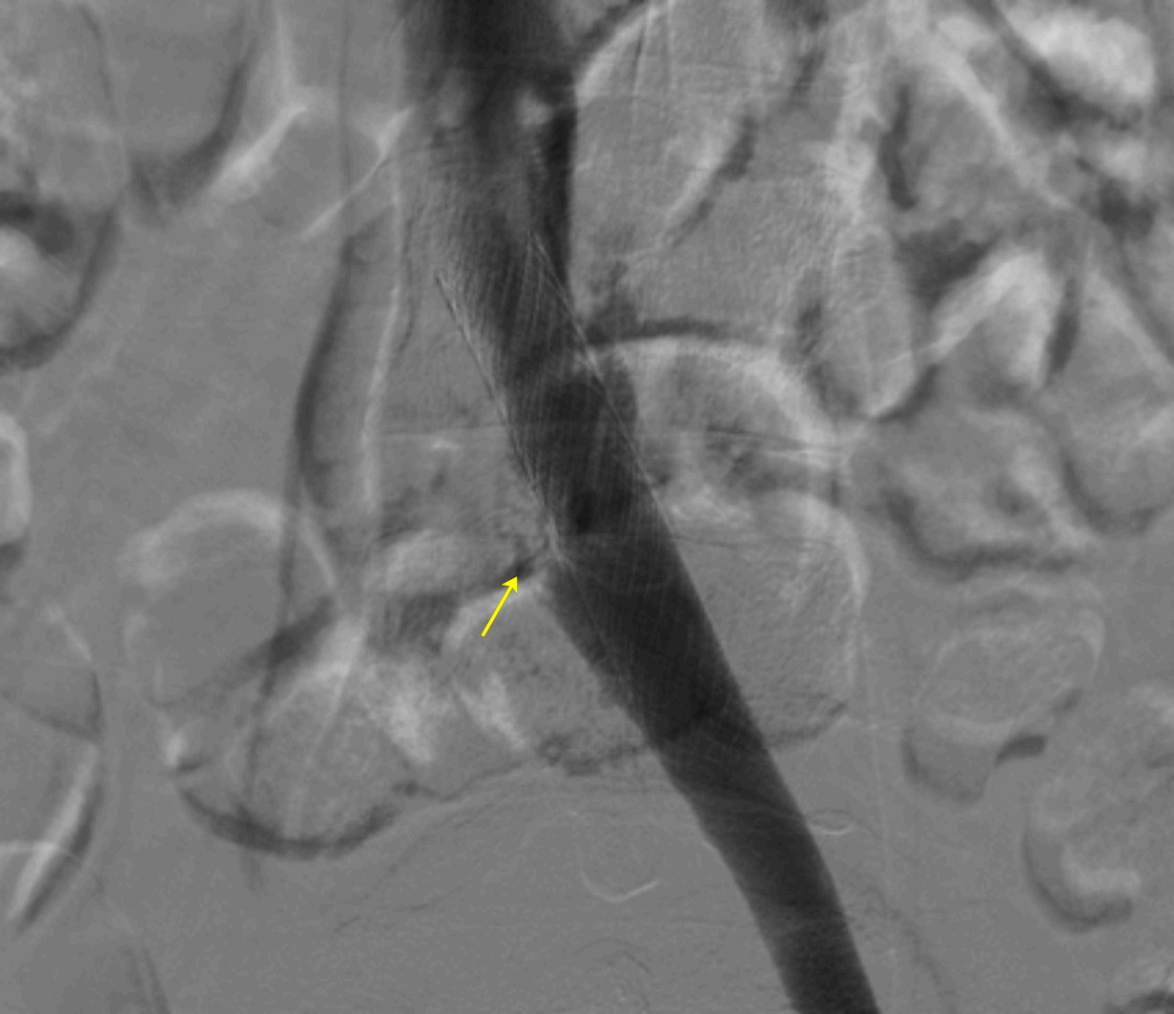
Control angiography after angioplasty and implantation of a Wallstent (Boston Scientific Corporation) in the left common iliac vein (LCIV) with satisfactory results (arrow).

**Figure 5:**
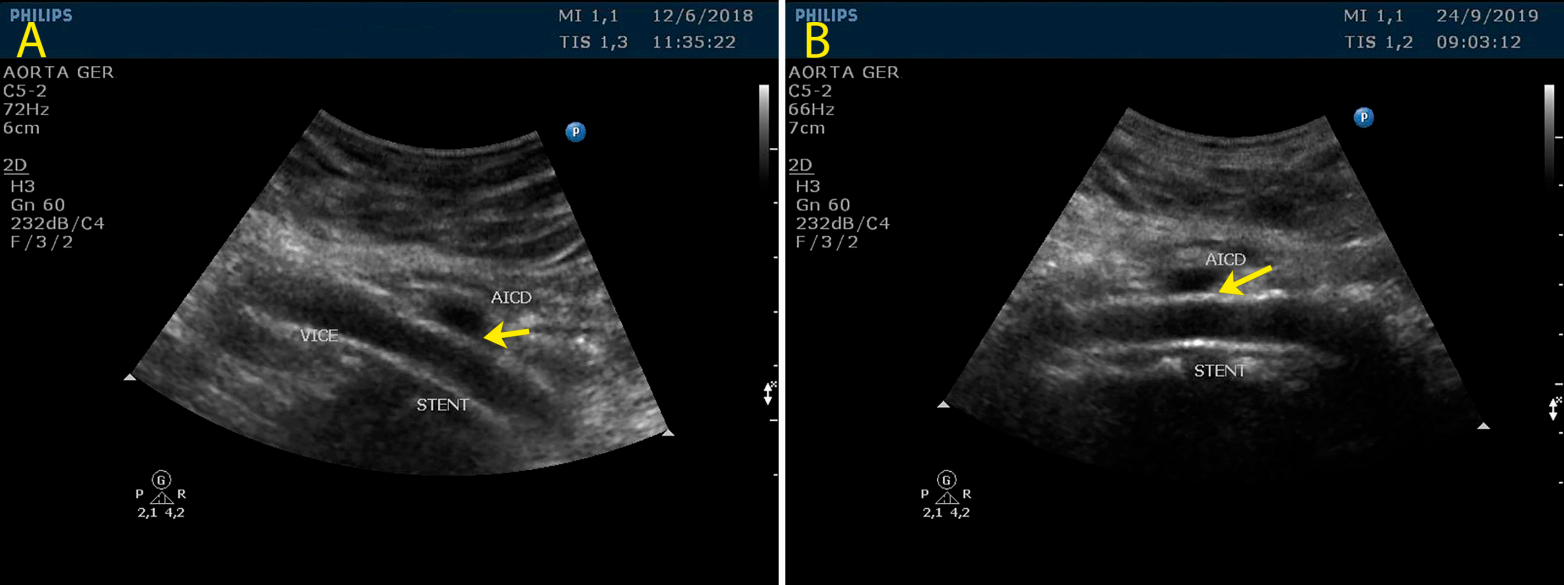
Control ultrasound in mode B showing the patent stent in the left common iliac vein (LCIV) and an absence of extrinsic compression: (A) 3 months after the procedure and (B) 18 months afterwards.

After the procedure, the patient was transferred to the intensive care unit (ICU), serial laboratory tests were conducted and she was monitored for bleeding. Control phlebography was conducted every 24 hours. 72 hours after fibrinolysis, symptoms had improved and control phlebography showed venous recanalization of the iliac-femoropopliteal segment and stenosis of the LCIV characteristic of May-Thurner Syndrome (Figure 3). In response to these images, LCIV angioplasty was performed using a 12x40 mm Mustang balloon catheter (Boston Scientific Corporation, Massachusetts, United States), with satisfactory phlebographic results (Figure 4). The patient was discharged from hospital asymptomatic 5 days after admission, showing improvements in her condition. She was prescribed 15 mg rivaroxaban every12 hours for 21 days, followed by 20 mg per day for 6 months. At a follow-up consultation 10 days later, she was already free from edema and pain and has since been asymptomatic for 24 months. She was not examined with DUS after the procedure because phlebography and physical examination had already provided evidence of recanalization of the iliac veins and deep veins of the leg. Control DUS examinations were conducted at 3, 6, and 18 months, yielding images showing the stent patent and the LCIV free from compression or narrowing, with a discretely reduced caliber, thickened walls, and blood flow present (Figure 5).

## DISCUSSION

Patients with FFVT in the lower limbs are at increased risk of PTE because of instability of the thrombus. Norris et al.^5^ conducted a retrospective analysis of the risk of PTE in patients with iliofemoral DVT with free-floating thrombi and concluded that the risk is significant despite treatment with heparin.

There are not yet any guidelines in the literature specifically for FFVT, and different approaches diverge greatly in terms of treatment, ranging from full anticoagulation,^10^ fibrinolytic treatment,^11^ and vena cava filters (VCF)^10^ to more invasive interventions such as surgical interruption^6^ with ligature of the venous system. In general, presence of FFVT is considered an absolute indication for rest,^12^ but there are no controlled studies that prove the need for this or the duration of immobilization.

Anticoagulation is the standard treatment for DVT in lower limbs, but in FFVT cases, medication alone may be insufficient to prevent displacement of the thrombus and, consequently, pulmonary thromboembolism (PTE).

Surgical procedures are one option, but the high mortality rate associated with ligature of the IVC prompted development of techniques for partial occlusion of larger veins or use of clips.^13^

Casian et al.^6^ claim that conservative management of FFVT, reserving more aggressive interventions for cases complicated with primary or recurrent VTE, is a questionable approach and even constitutes a risk for the patient, which would justify invasive treatment, despite the high perioperative mortality. In a series of thirteen cases described by Casian et al.^6^, they performed plication or ligation of deep veins in all cases of FFVT proximal of the confluence of the superficial-deep femoral vein, plication of the common femoral vein combined with partial thrombectomy, and plication of the common iliac vein. No cases of clinically significant primary or recurrent VTE were detected.

Thrombectomy with removal of the thrombotic mass via a Fogarty catheter is another option since, in addition to preventing severe postthrombotic syndrome, it reduces the risk of PTE. Fibrinolytic treatment also reduces postthrombotic syndrome with dissolution of the thrombus, but it is associated with hemorrhagic complications.^14^ In turn, a VCF is advisable when there are anticoagulant contraindications or complications. However, VCFs are associated with a high risk of VTE in cases of extensive floating thrombi involving the iliac veins and vena cava.^15^

Xue et al.^16^ assessed the safety and efficacy of catheter-directed thrombolysis and stenting for treatment of iliac vein compression syndrome with iliofemoral DVT in 61 patients. They fitted a VCF in 28 patients and used 68 stents. The pressure gradient across the iliac vein stenosis reduced significantly after the procedure and there were 66.7 and 61.6% reductions in the thigh and calf circumferences, respectively. They did not observe large hematoma, stent migration, or acute thrombosis during the procedures. They concluded that thrombolysis and implantation of a stent resulted in good patency and vein function in these cases after 5 years’ follow-up. Nevertheless, more evidence is needed to establish the benefits over the longer term.^16^

Endovascular treatment motivated by worsening of the patient’s symptoms after conservative management was effective in the case described, carefully following our institution’s protocol for catheter-directed thrombolysis. Good results were achieved with the treatment chosen, considering the improved clinical status confirmed by phlebography showing venous recanalization.

The posterior discovery of May-Thurner Syndrome confirms the importance of this disease in thrombotic events associated with compressive syndromes involving the iliocaval segment. Definitive treatment with angioplasty and a self-expanding stent has proven effective and with low rates of relapse and new thrombotic events over the short and medium term.

## CONCLUSIONS

Although several treatment options for cases of FFVT in lower limb deep veins are described in the literature, there are not yet any specific guidelines. We believe that the choice of fibrinolysis, following the institutional protocol, is one option for extensive DVT in lower limbs, including in the present case with free-floating thrombus. This approach has a role to play in patients with severe symptomology and long life expectancy.
