# Supplementary material for: Free-floating thrombus in lower limb deep veins and May-Thurner syndrome: case report
Source: J Vasc Bras. 2020 Nov 16;19:e20200075. doi: 10.1590/1677-5449.200075 (PMC8218010; doi:10.1590/1677-5449.200075)
Supplement: Translation

# Translation: Trombo flutuante em veias profundas de membro inferior e síndrome de May-Thurner: relato de caso

**Authors:** Helio Bergantini, Selma Regina de Oliveira Raymundo, Daniel Gustavo Miquelin, Gleison Juliano da Silva Russeff, Desirée Francini del Frari Silva, Gabriela Leopoldino da Silva, Amabile Oficiati de Carnevale Galeti, Matheus Pietraroia dos Santos

**Affiliations:** 1 Faculdade de Medicina de São José do Rio Preto – FAMERP, São José do Rio Preto, SP, Brasil.; 2 Hospital Austa de São José do Rio Preto, São José do Rio Preto, SP, Brasil.

**Keywords:** trombose venosa, anticoagulantes, fibrinólise, embolia pulmonar

## Abstract

O trombo flutuante no sistema venoso profundo manifesta elevado potencial de embolização pulmonar. Pode também ser encontrado em pacientes com trombose venosa superficial (TVS) com extensão para uma veia profunda. Os tratamentos descritos na literatura, ainda sem critérios definidos, variam desde anticoagulação e tratamentos fibrinolíticos com implantes de filtros de veia cava, trombectomias abertas ou com dispositivos endovasculares até condutas mais invasivas como a interrupção cirúrgica com ligadura do sistema venoso. Apresentamos o caso de uma paciente com trombose venosa profunda extensa, acometendo o território ilíaco-fêmoro-poplíteo com um trombo flutuante estendendo-se da veia ilíaca comum esquerda até a veia cava inferior. O tratamento foi realizado com terapia fibrinolítica com um cateter multiperfurado, associado a anticoagulação com heparina e a controles angiográficos diários. Ao final do tratamento, foi identificada uma estenose significativa na veia ilíaca comum esquerda, sendo realizada angioplastia com implante de stent.

## INTRODUÇÃO

Há mais de três décadas, o tromboembolismo venoso (TEV) foi estimado como o terceiro evento cardiovascular agudo mais comum no mundo com incidência anual global nos Estados Unidos entre 1 e 2 por 1.000 pessoas^1^^-^3. Trombos localizados no sistema venoso profundo dos membros inferiores (MMII) constituem a maior fonte de embolização, principalmente as veias ilíacas, femorais e poplíteas^4^. Também a síndrome de May-Thurner tem importância na gênese de doenças venosas profundas.

O trombo venoso flutuante (TVF) consiste em uma forma particular de trombose venosa profunda (TVP) com alto potencial de embolização pulmonar^5^^,^^6^. Um TVF exibe um movimento de oscilação contínuo da massa trombótica não aderida à parede venosa em regiões de confluências venosas maiores: femoral superficial-profunda, safeno-femoral, ilíaca interna-externa, ilio-caval^6^. O TVF também pode estar presente em pacientes com trombose venosa superficial (TVS) isolada^7^, sendo possível se estender para uma veia profunda e, ainda, ser uma complicação do *laser* endovenoso para tratamento de veias safenas insuficientes^8^.

Estudos sobre TVF são escassos e divergem em aspectos como mortalidade, prevalência e localização predominante dos trombos. Na pesquisa desenvolvida por Voet e Afschrift^9^, em 44 casos de TVP proximal, a prevalência de TVF foi de 18% com a seguinte distribuição: 38% na junção safeno-femoral, 26% na junção da veia safena parva e 15% na veia ilíaca externa. Os autores constataram com ultrassom Doppler (USD) que, após 3 meses de tratamento anticoagulante, 87% dos trombos despareceram, independentemente da sua localização. Norris et al.^5^, por sua vez, constataram, em 78 pacientes hospitalizados com TVP iliofemoral diagnosticados por venografia, uma notável diferença no risco de embolização pulmonar, confirmada por cintilografia pulmonar ventilação/perfusão realizada 10 dias após venografia entre trombos oclusivos (5,5%) e livres flutuantes (60%). Todos os pacientes foram tratados com heparina. Já Yamaki et al.^10^ documentaram uma taxa de 1,7% de trombos flutuantes em 427 pacientes diagnosticados com TVP, localizados majoritariamente (71,4%) no segmento fêmoro-poplíteo. O fato do TVF se formar tipicamente nas confluências de grandes vasos, aliado à sua maior instabilidade, confere esse potencial elevado de complicaçôes^4^^-^6.

No que se refere à abordagem terapêutica, existem divergências na literatura. Alguns estudos sugerem apenas anticoagulação devido à autólise dos trombos flutuantes e repouso^9^^,^^10^. Outros indicam abordagem cirurgica^6^. Apresentamos um caso de TVF em veia ilíaca comum esquerda (VICE) tratado com procedimento endovascular com fibrinólise, angioplastia e implante de stent.

## DESCRIÇÃO DO CASO

Paciente de 47 anos, sexo feminino, admitida com dor intensa e edema em membro inferior esquerdo (MIE) há um dia. Ao exame físico, detectou-se edema nesse membro desde a coxa, empastamento, pletora venosa e sinais de Homans e Bancroft. O USD evidenciou veias ilíacas comum e externa esquerda com calibres aumentados, fluxo ausente e material hipoecoico intraluminal indicando TVP aguda. As veias femorais, poplítea, tibiais e fibulares eram compressíveis e com fluxo normal, não evidenciando presença de trombos (Figura 1).

**Figura 1:** Ultrassom Doppler (USD) mostrando: (A) veia ilíaca comum esquerda (VICE) com calibre aumentado e fluxo ausente, com material hipoecoico intraluminal indicando trombose venosa profunda (TVP) aguda e compressão pela artéria ilíaca comum direita (AICD); e (B) veia femoral superficial esquerda (VFSE) com calibre normal, fluxo presente e ausência de trombos.

Iniciou-se anticoagulação com heparina de baixo peso molecular (HBPM) em dose plena. Após dois dias do tratamento, a paciente manifestou piora do edema, e optou-se por tratamento trombolítico.

A flebografia realizada por meio de punção da veia poplítea esquerda guiada por USD com a utilização de cateter 6Fr Bernstein (AngioDynamics, Inc., Nova Iorque, EUA) possibilitou visualizar a progressão da trombose para as veias femorais e poplítea, que não existia quando realizado USD no dia da internação, como pode ser observado na Figura 1. Mostrou, ainda, um trombo flutuante na veia cava inferior (VCI) (Figura 2). Para a realização da fibrinólise, foi posicionado um microcateter multifenestrado Fountain (Merit Medical Systems, Inc., Utah, EUA) até a VICE e injetado 10 mg em *bolus* do fibrinolítico Alteplase. O cateter foi mantido no local para a infusão contínua do fibrinolítico na dose de 0,01 mg/kg/hora. Acrescentou-se heparina endovenosa, em infusão contínua, simultaneamente ao uso do fibrinolítico.

**Figura 2:** Flebografia mostrando: (A) progressão da trombose para as veias femorais e poplítea; (B) trombose extensa das veias ilíacas esquerda e da veia cava inferior (VCI); e (C) presença de trombo flutuante em VCI (seta).

Na sequência do procedimento, a paciente foi encaminhada à unidade de terapia intensiva (UTI), com exames laboratoriais seriados e monitorização de sangramentos. O controle flebográfico foi realizado a cada 24 horas. Após 72 horas de fibrinólise, verificou-se melhora dos sintomas, e a flebografia de controle revelou recanalização venosa no segmento ilíaco-fêmoro-poplíteo e presença de estenose em VICE, característica da síndrome de May-Thurner (Figura 3). Diante das imagens, efetuou-se angioplastia de VICE, com cateter balão Mustang 12x40 mm (Boston Scientific Corporation, Massachusetts, EUA), seguido de implante de Wallstent 14x40 mm (Boston Scientific Corporation) em VICE no local da estenose, obtendo-se resultado flebográfico satisfatório (Figura 4). A paciente recebeu alta hospitalar 5 dias depois da admissão assintomática, notando-se evolução favorável do quadro. Utilizou-se rivaroxabana 15 mg de 12/12 horas durante 21 dias e, posteriormente, 20 mg ao dia pelo período de 6 meses. No retorno, 10 dias subsequentes, não apresentou edema e/ou dor e encontra-se assintomática após 24 meses. Não foi realizado USD pós-procedimento pois os exames flebográfico e físico já evidenciavam recanalização das veias ilíacas e veias profundas da perna. USD de controle foram realizados em 3, 6 e 18 meses e mostraram imagem de stent pérvio, VICE sem compressão ou estreitamento, com calibre discretamente reduzido, paredes espessadas e fluxo presente (Figura 5).

**Figura 3:** Flebografia de controle após 72 horas de fibrinólise evidenciou: (A) recanalização da veia cava inferior (VCI) e veia ilíaca comum esquerda (VICE); (B) recanalização da veia femoral superficial; e (C) presença de compressão extrínseca de VICE, característica da síndrome de May-Thurner (seta).

**Figura 4:** Controle angiográfico após angioplastia e implante de Wallstent (Boston Scientific Corporation) em veia ilíaca comum esquerda (VICE) com resultado satisfatório (seta).

**Figura 5:** Controle ultrassonográfico em modo B evidenciando stent pérvio na veia ilíaca comum esquerda (VICE) e ausência de compressão extrínseca: (A) 3 meses após procedimento e (B) 18 meses após.

## DISCUSSÃO

Os pacientes com TVF de MMII exibem um risco maior de TEP por causa da instabilidade do trombo. Norris et al.^5^ avaliaram retrospectivamente o risco de TEP em pacientes com TVP iliofemoral com trombo livre-flutuante e concluíram que esse risco é significante, apesar do tratamento com heparina.

Ainda não existe na literatura uma diretriz específica para TVF, e as condutas são bastante divergentes quanto à terapêutica: desde anticoagulação plena^10^, tratamentos fibrinolíticos^11^ e implantes de filtros de veia cava (FVC)^10^ até condutas mais invasivas como a interrupção cirúrgica^6^, com ligadura do sistema venoso. A presença de TVF, em geral, é considerada uma indicação absoluta para repouso^12^, mas não há estudos controlados que comprovem tal necessidade e qual o período de imobilização.

A anticoagulação é o tratamento padrão em TVP de membros inferiores, entretanto, nos casos de TVF, apenas a medicação pode não ser suficiente para prevenir o deslocamento do trombo e, como consequência, o tremboembolismo pulmonar (TEP).

Procedimentos cirúrgicos consistem em uma alternativa, mas a taxa de mortalidade alta associada com a ligadura de VCI resultou em técnicas para oclusão parcial das veias maiores ou aplicação de clipes^13^.

Casian et al.^6^ defendem que o tratamento conservador para TVF e somente manejo mais agressivo para casos complicados com TEV primário ou recorrente representam uma abordagem duvidosa e até um risco ao paciente, o que justificaria o tratamento invasivo, apesar da alta mortalidade periprocedimento. Em treze casos descritos por eles, fizeram plicatura ou ligadura de veias profundas em todos casos de TVF proximais à confluência venosa femoral superficial/profunda, plicatura de veia femoral comum associada à trombectomia parcial e plicatura de veia ilíaca comum. Nenhum caso de TEV primário ou recorrente clinicamente significante foi detectado.

A trombectomia com remoção da massa trombótica com cateter de Fogarty constitui uma alternativa, pois, além de prevenir uma síndrome pós-trombótica severa, diminui o risco de TEP. O tratamento fibrinolítico também reduz a síndrome pós-trombótica com a dissolução do trombo; todavia, está associado a complicações hemorrágicas^14^. O FVC, por sua vez, é aconselhável quando há contraindicação ou complicações dos anticoagulantes. Contudo, está associado a alto risco de TEV nos casos de trombos flutuantes extensos envolvendo veias ilíacas e veia cava^15^.

Xue et al.^16^ avaliaram a segurança e eficácia da trombólise dirigida por cateter e implante de stent no tratamento da síndrome da compressão da veia ilíaca com TVP iliofemoral de 61 pacientes. Implantou-se FVC em 28 deles, e foram usados 68 stents. O gradiente de pressão ao redor da estenose da veia ilíaca diminuiu significativamente após o procedimento e houve redução das circunferências da coxa e panturrilha de 66,7 e 61,6%, respectivamente. Não se observaram hematoma grande, migração do stent ou trombose aguda durante o procedimento. Os autores concluem que a trombólise e implantação de stent nesses casos mostraram boa perviedade e função da veia após 5 anos de seguimento. No entanto, mais evidências são necessárias para estabelecer os benefícios em prazos mais longos^16^.

O tratamento endovascular motivado pela piora dos sintomas da paciente após a abordagem conservadora foi eficaz no caso descrito, seguindo-se de forma criteriosa o protocolo de trombólise cateter-dirigido da nossa instituição. Chegou-se a bons resultados com a terapêutica utilizada, haja vista a melhora do quadro clínico comprovada na flebografia evidenciando recanalização venosa.

A posterior descoberta da síndrome de May-Thurner corrobora a importância da doença em eventos trombóticos associados às síndromes compressivas em segmento cavo-ilíaco. O tratamento definitivo com angioplastia e stent autoexpansível revelou-se eficiente e com baixa recidiva de novo evento trombótico a curto e médio prazos.

## CONCLUSÃO

Embora várias opções de tratamento nos casos de TVF em veias profundas dos MMII sejam descritas na literatura, ainda não existe uma diretriz específica. Acreditamos que a opção pela fibrinólise, seguindo o protocolo institucional, possa ser uma alternativa para TVP extensa de MMII, incluindo o presente caso com trombo flutuante associado. Ressalta-se, por fim, o papel da conduta em questão em pacientes com sintomatologia exuberante e elevada expectativa de vida.
